# MicroRNA Profiling of HL-1 Cardiac Cells-Derived Extracellular Vesicles

**DOI:** 10.3390/cells10020273

**Published:** 2021-01-30

**Authors:** Serena Silvestro, Agnese Gugliandolo, Luigi Chiricosta, Francesca Diomede, Oriana Trubiani, Placido Bramanti, Jacopo Pizzicannella, Emanuela Mazzon

**Affiliations:** 1 IRCCS Centro Neurolesi “Bonino-Pulejo”, 98124 Messina, Italy; serena.silvestro@irccsme.it (S.S.); agnese.gugliandolo@irccsme.it (A.G.); luigi.chiricosta@irccsme.it (L.C.); placido.bramanti@irccsme.it (P.B.); 2 Department of Innovative Technologies in Medicine & Dentistry, University “G. d’Annunzio”, Chieti-Pescara, Via dei Vestini, 31, 66100 Chieti, Italy; francesca.diomede@unich.it (F.D.); oriana.trubiani@unich.it (O.T.); 3 Ss. Annunziata” Hospital, ASL 02 Lanciano-Vasto-Chieti, 66100 Chieti, Italy; jacopo.pizzicannella@unich.it

**Keywords:** HL-1 cardiac cells, extracellular vesicles, microRNAs, next generation sequencing

## Abstract

HL-1 is a cell line that shows a phenotype similar to adult cardiomyocytes. All major cardiac cell types release extracellular vesicles (EVs) that emerge as key mediators of intercellular communication. EVs can mediate intercellular cross-talk through the transfer of specific microRNAs (miRNAs). MiRNAs are known to play important regulatory roles during tissue differentiation and regeneration processes. Furthermore, miRNAs have recently been shown to be involved in the proliferation of adult cardiomyocytes. In this context, the purpose of this study was to analyze the transcriptomic profile of miRNAs expressed from HL-1 cardiac muscle cell-derived EVs, using next generation sequencing (NGS). Specifically, our transcriptomic analysis showed that the EVs derived from our HL-1 cells contained miRNAs that induce blood vessel formation and increase cell proliferation. Indeed, our bioinformatics analysis revealed 26 miRNAs expressed in EVs derived from our HL-1 that target genes related to cardiovascular development. In particular, their targets are enriched for the following biological processes related to cardiovascular development: heart morphogenesis, positive regulation of angiogenesis, artery development, ventricular septum development, cardiac atrium development, and myoblast differentiation. Consequently, EVs could become important in the field of regenerative medicine.

## 1. Introduction

In recent studies, the paracrine effects of mesenchymal stem cells are considered to be the main reason for their regenerative potential; therefore, the focus has been on extracellular vesicles (EVs) and the bioactive molecules they contain [1,2]. Cell-free strategies using conditioned mediums [3,4] or EVs [5] demonstrate that these approaches might be beneficial when considering the risk factors associated with stem cell use. Recent evidence shows that EVs are generating significant interest in the cardiovascular field due to their wide range of potential applications [6]. EVs are systems of intercellular communication that interact with target cells through binding to the cell surface receptors and releasing their contents into the recipient cell’s plasma membrane [7,8]. The EVs contain several lipids, proteins, a pool of soluble cytokines, and nucleic acids such as mRNAs and microRNAs (miRNAs) [9].

Specifically, miRNAs belong to the small non-coding RNA family and they are involved in the negative regulation of gene expression at the post-transcriptional level. MiRNAs act in a binding manner to mRNA complementary sequences, inhibiting translation and/or inducing their degradation [10]. MiRNAs can regulate multiple genes, and the same gene can be regulated by multiple miRNAs [11]. MiRNAs are highly conserved across species and contribute to cellular proliferation, differentiation, and apoptosis processes [12]. To date, it is known that miRNAs play an important role in the proliferation and differentiation processes of pacemaker cells, cardiac myocytes, vascular smooth muscle cells, and endothelial cells [13]. Therefore, EVs represent an interesting therapeutic approach in the field of heart tissue regeneration.

The HL-1 is a cardiac muscle cell line, which maintains a pattern of gene expression similar to normal atrial cardiac myocytes in adult mice. HL-1 cells retain the ability to proliferate without returning to an embryonic phenotype. Moreover, HL-1 cells maintain differentiated cardiac morphological, biochemical, and electrophysiological properties [14]. 

In this study, through next generation sequencing (NGS) analysis, we evaluated the transcriptomic profile of miRNAs expressed in EVs derived from the HL-1 cardiac muscle cell line. 

## 2. Materials and Methods

### 2.1. HL-1 Mouse Cell Culture Establishment

HL-1 is a mouse cardiac muscle cell line purchased from Sigma-Aldrich (cat. n. SCC065, Merck, Milan, Italy). HL-1 cells were manipulated as reported by manufacturer instructions. Cells were cultured in a Claycomb medium (cat. n. 51800C, Sigma-Aldrich, St. Louis, MO, USA) supplemented with 10% fetal bovine serum (FBS) (cat. n. TMS-016, Sigma-Aldrich), 1% penicillin/streptomycin (cat. n. P4333, Sigma-Aldrich, St. Louis, MO, USA), Norepinephrine 10 mM (cat. n. A0937, Sigma-Aldrich, St. Louis, MO, USA), and L-Glutamine 2 mM (cat. n.A7506, Sigma-Aldrich, St. Louis, MO, USA). Before culturing the cells, tissue culture flasks (T75, Corning, Merck) were coated with 3 mL of gelatin/fibronectin (cat. n. G9391/F1141, Sigma-Aldrich, St. Louis, MO, USA) for 1 h in a humidified atmosphere at 37 °C and 5% CO_2_.

### 2.2. HL-1 Morphological Analyses

The HL-1 cardiac muscle cell line was observed with the inverted light microscopy DM IL (Leica Microsystem, Milan, Italy). Moreover, to better evaluate the morphological features present, HL-1 cells grown on gelatin/fibronectin-coated coverslips were fixed with 2.5% paraformaldehyde in phosphate buffered saline (PBS) (pH 7.2) for 15 min at 4 °C and permeabilized with 0.1% Triton X-100 for 3 min at room temperature [15]. Samples were incubated with the Alexa Fluor 488 phalloidin green fluorescence conjugate (1:400, Molecular Probes, Eugene, OR, USA) for 1 h, to highlight the cytoskeleton actin. Then, after washing, cells were incubated with TOPRO (1:200, Molecular Probes) for 1 h at 37 °C to mark cell nuclei. Samples were put facing down on glass slides and mounted with Prolong antifade (Molecular Probes) [16]. Cultures were examined on a Zeiss LSM800 (Zeiss, Jena, Germany) confocal laser scanning microscope equipped with an inverted Zeiss Axiovert 200 microscope furnished with a Plan Neofluar oil-immersion objective (63×/1.3 NA). 

### 2.3. EVs Extraction from HL-1 Cardiac Muscle Cells

The conditioned medium (CM) was recovered from the HL-1 cell culture (with a volume of 10 mL) after 48 h of incubation. The CM was centrifuged at 3000× *g* for 15 min to eliminate suspension cells and debris. For EV extraction, the ExoQuick TC commercial agglutinant (System Biosciences, Euroclone SpA, Milan, Italy) was used. Briefly, 2 mL of ExoQuick TC solution was added to 10 mL of CM. The mix was incubated overnight at 4 °C without rotation; one centrifugation step was performed at 1500× *g* for 30 min to sediment the EVs, and the pellets were re-suspended in 200 μL of PBS [17]. The detection of the whole homogenate proteins of the EVs was used as a confirmation of the presence of EV release in HL-1.

### 2.4. Atomic Force Microscopy (AFM) Measurements

In order to evaluate the surface morphology of the EVs, atomic force microscopy (AFM) measurements were performed using a Multimode 8 Bruker AFM microscope with a Nanoscope V controller (Bruker AXS, Marne La Vallee, France). It is worth highlighting that AFM analyses were performed to visualize the prevailing smallest exosomal objects, since it is very difficult to visualize very irregular micrometric surfaces such as those that one could expect for aggregated micro-vesicles. Nevertheless, several authors have already used this technique to visualize EVs, thus exploiting the mild experimental conditions under which it is possible to visualize them and avoiding the high vacuum of transmission electron microscopy measurements by using structural–mechanical characterization of nanoparticle exosomes in human saliva, using correlative AFM, FESEM, and force spectroscopy [18]. A silicon cantilever and a RTESPA-300 tip (with a spring constant of 40 N/m and a resonant frequency of 300 Hz) were used for tapping in the air mode. The specimen was prepared by dropping a solution of EVs on a SiO_2_ wafer, followed by air drying at 37 °C for 1 h. The solutions of EVs dropcasted onto SiO_2_ water had a different concentration because we wanted to avoid the formation of large aggregates, as previously described [19].

### 2.5. EV Protein Extraction for Western Blot Analysis

EVs derived from HL-1 and whole cell lysate of HL-1 (used as control) were re-suspended in an Radioimmunoprecipitation assay (RIPA) cold hypotonic lysis buffer (1× PBS, 1% Igepal, 0.5% sodium deoxycholate, 0.1% sodium dodecyl sulphate (SDS), 10 µg/mL phenylmethylsulfonyl fluoride (PMSF) and 10 μl/mL of Protease Inhibitor Cocktail (Sigma-Aldrich, Milan, Italy). The level of recovered protein was measured spectrometrically according to the manufacturer’s instructions using the Bio-Rad (Hercules, CA, USA) Protein Assay (detergent compatible). Proteins were separated on sodium dodecyl sulfate–polyacrylamide mini gels and transferred onto polyvinylidene difluoride (PVDF) membranes (Immobilon-P Transfer membrane, Millipore, Merck), blocked with PBS containing 5% non-fat dried milk (PM) for 45 min at room temperature, and subsequently probed at 4 °C overnight with specific antibodies, CD9 (1:2000; Novus Biologicals, Milan, Italy), CD63 (1:500; Santa Cruz Biotechnology Inc., Santa Cruz, CA, USA), and anti-Calnexin (1:2000; Abcam, Prodotti Gianni, Milan, Italy). Horseradish peroxidase (HRP)-conjugated goat anti-mouse IgG was incubated as a secondary antibody (1:2000; Santa Cruz) for 1 h at room temperature [20]. The relative expression of protein bands was visualized using an enhanced chemiluminescence system (Luminata Western HRP Substrates, Millipore), and protein bands were acquired and quantified with the ChemiDoc MP System (Bio-Rad, Hercules, CA, USA), and a computer program UVIband-1D gel analysis software (Uvitec, Cambridge, UK), respectively [21]. The statistical analysis was carried out on three repeated blots performed on separate experiments.

### 2.6. Small RNA Extraction and miRNA Library Preparation

The library preparation was carried out according to the TruSeq Small RNA Library Prep Kit (Illumina, San Diego, CA, USA) following manufacturer instructions. Briefly, adapters were ligated to the 3’ and 5’ ends of the sample, and a reverse transcription, followed by amplification, created cDNA constructs. The cDNA constructs were then purified using a Tris Borate EDTA (TBE) gel. Finally, the library was sequenced on the MiSeq Instrument (Illumina).

### 2.7. MiRNA Analysis through Bioinformatics Pipeline

**Figure 1:**
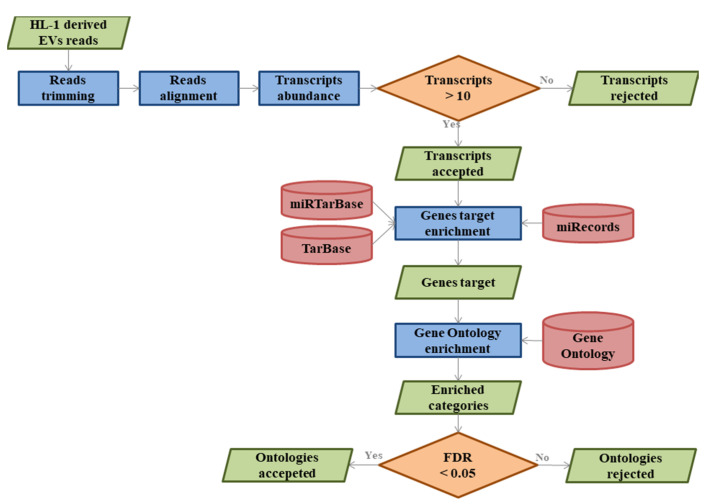
Flowchart of the bioinformatics analysis implemented in the study.

In the flowchart in Figure 1, we provided the workflow of the bioinformatics procedure. The raw data obtained in FASTQ format by the sequencer were checked for quality using FastQC software while cutadapt [22] was used to remove the bases with low quality and trim the adapter sequences (Reads trimming step). A custom up-to-date version of the Mus Musculus genome was generated using the spliced transcripts alignment to a reference (STAR) RNA-Seq aligner [23] (version 2.7.6a) using the “—twopassMode Basic” parameter to increase the mapping change of the reads. The genome was built on GRCm38 (version downloaded by GENCODE) archive (ftp://ftp.ebi.ac.uk/pub/databases/gencode/Gencode_mouse/release_M25/). The reads were then aligned by STAR (via read alignment) on this genome and the features annotated as miRNA were then counted through the htseq-count Python package (Transcripts abundance step) [24]. Only the miRNAs counted at least 10 times in each experiment were kept in the analysis. In order to provide a biological involvement to the miRNAs, we used the multiMiR package of Bioconductor (Genes target enrichment step) [25]. In particular, we focused on miRecords [26], miRTarBase [27] and TarBase [28] databases that collect validated interactions between miRNAs and their targets. The found target genes were then enriched with the biological process category of gene ontology [29] with Panther [30] (Gene Ontology enrichment step). The categories where False Discovery Rate (FDR) > 0.05 were rejected.

## 3. Results

### 3.1. HL-1 Morphological Features and EVs Characterization

**Figure 2:**
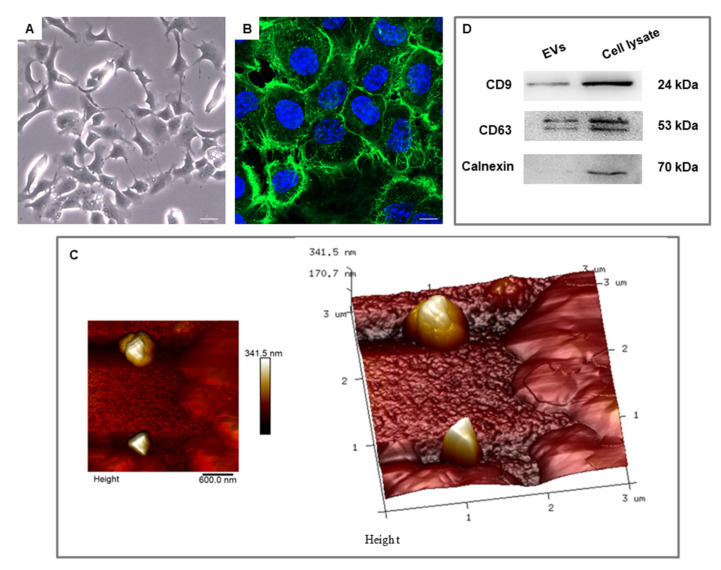
HL-1 cell culture and derived extracellular vesicles (EVs). (**A**) HL-1 morphology observed under inverted light microscopy (10×). Scale bar: 0.2 µm. (**B**) Immunofluorescence staining of HL-1 observed under confocal microscopy (63×). The nuclei were highlighted in blue, while the actin of the cytoskeleton was highlighted in green. Scale bar: 10 µm. (**C**) EVs were analyzed under atomic force microscopy to evaluate their morphological aspect. (**D**) Western Blot experiments of CD9, CD63, and Calnexin performed on cell lysate used as controls and on a fraction of the EVs.

The HL-1 cardiac muscle cell line showed a rounded morphology when observed with light microscopy (Figure 2A). Under confocal microscopy, they also exhibited a circular morphology, with evident central nuclei stained in blue. Cytoskeleton actin, stained in green, was arranged at the peripheral area at the cytoplasmic level (Figure 2B). EVs were analyzed by AFM, demonstrating the presence of globular EVs of different dimensions with a smooth surface (Figure 2C).

Western blot analysis performed on the EVs derived from HL-1 cell culture showed a positive trend for CD9 and CD63 molecules and a negative expression for Calnexin, excluding the presence of a cellular compartment to a fraction of the EVs (Figure 2D and Supplementary Materials Figure S1). The present protein identification indicates the successful isolation of the EVs.

### 3.2. Analysis of miRNA Expressed in HL-1 EVs

The bioinformatics analysis reveals 26 miRNAs expressed in HL-1 EVs, shown in Table 1. Interestingly, we can observe a median distribution of 57.33, while the miRNAs *mir143* (mean expression 1390.67), *mir99b* (mean expression 412.00), *mir22* (mean expression 335.33) and *mir182* (mean expression 328.00) are very highly expressed and are outliers in the distribution array.

**Figure 3:**
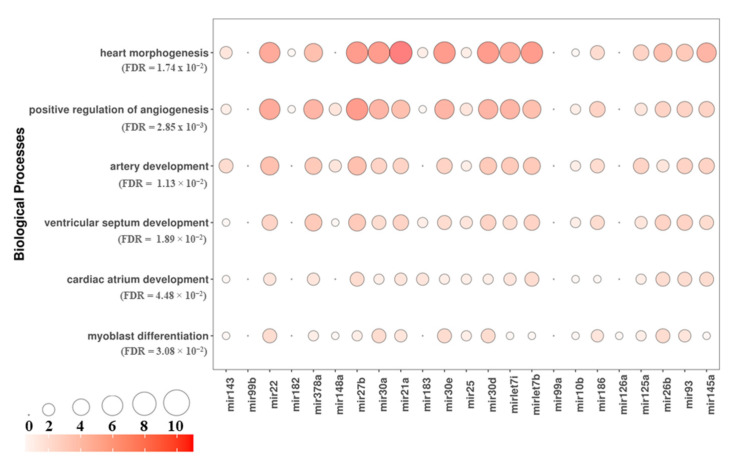
Bubbleplot of the biological processes related to myogenesis that characterizes the target genes of the miRNAs expressed in HL-1 vesicles. For each of the six biological processes that are showed on the rows, the significance value obtained with Panther (FDR) was highlighted and the number of genes that are targeted by the miRNAs in each column were shown. The miRNAs are sorted by expression level (from the left to the right). The white-red scale and the circles size show the number of target genes in the process (in order from the least to the most).

In order to characterize the role of the miRNAs found in HL-1 EVs, we collected their target genes through miRecords, miRTarBase, and TarBase databases. We collected 262 targets that were enriched with the biological process in terms of gene ontology. We focused on the processes related to cardiovascular development such as heart morphogenesis (FDR = 1.74 × 10^−2^), positive regulation of angiogenesis (FDR = 2.85 × 10^−3^), artery development (FDR = 1.13 × 10^−2^), ventricular septum development (FDR = 1.89 × 10^−2^), cardiac atrium development (FDR = 4.48 × 10^−2^), and myoblast differentiation (FDR = 3.08 × 10^−2^). The bubbleplot in Figure 3 shows the most to the least expressed miRNAs when read from left to right, and the amount of genes in those categories escalating from small white circles to large red circles. Interestingly, some of the miRNAs that were less frequently expressed (such as *mir125a*, *mir26b*, *mir93* and *mir145a*) target more genes than the most expressed miRNAs, such as *mir182* and *mir99b*.

## 4. Discussion

Myocardiogenesis is a process that determines the role of cardiac cells during embryogenesis. Both the acquisition and the maintenance of heart morphology can be mediated by epigenetic factors, such as miRNAs. The miRNAs act as important post-transcriptional regulators capable of modulating gene expression [31]. In our study, we analyzed the miRNAs contained in EVs derived from HL-1 cardiac muscle cells. These cells are a line of mouse cardiocytes capable of dividing continuously, maintaining a differentiated cardiac phenotype [32]. As demonstrated by our microscope images, HL-1 cells highlighted round shape morphological features (Figure 2A, B). AFM analysis showed that EVs presented a globular morphology with a smooth surface (Figure 2C). Moreover, our Western blot analysis showed that EVs possess a positivity for CD9 and CD63 (Figure 2D), specific membrane-associated proteins that are elevated in the EVs compared to their levels in the cells from which they are derived [33,34]. Moreover, they did not express Calnexin, excluding the impurity derived from other compartments to the EVs fractionally (Figure 2D). The results of the study showed that our EVs contained several miRNAs (*mir143*, *mir99b*, *mir22*, *mir182*, *mir378a*, *mir148a*, *mir27b*, *mir30a*, *mir21a*, *mir183*, *mir30e*, *mirlet7i*, *mir25*, *mir30d*, *mir92-1*, *mirlet7b*, *mir99a*, *mir10b*, *mir186*, *mir126a*, *mir301*, *mir125a*, *mir26b*, *mir451a*, *mir93* and *mir145a*), listed in Table 1. Our bioinformatics analysis showed that the targets of our set of miRNAs are specifically enriched for processes related to the regulation of angiogenesis and cardiac development, as illustrated in the bubbleplot (Figure 3). For this reason, we investigated the literature about each miRNA in order to highlight their role in cardiovascular development.

In particular, we found an enrichment in the biological processes related to heart morphogenesis and development. Specifically, *mir143,* the most expressed miRNA, was shown to be involved in promoting the growth and elongation of myocardial cells in zebrafish. Indeed, miR-143 through the modulation of adducin3 (add3) gene, which encodes an F-actin capping protein, was discovered to be essential in the morphogenesis of cardiac chambers [35]. These results agree with our bioinformatic analysis that shows that some of its targets fall under biological processes related to cardiac morphogenesis.

HL-1 EVs also contained *mir27b*, which Chinchilla A. et al. have shown to be highly expressed during embryogenic heart development. It was expressed differentially from the early stages of mouse ventricular chamber formation. Specifically, miR-27 exerts this functional role during myocardiogenesis by regulating Myocyte-Enhancer Factor 2c (*MEF2C*) expression levels in myogenic cells [36]. Wang Y. et al. showed that miR-27b targets the ALK5 gene, a receptor of transforming growth factor-beta 1 (TGF-β1), regulating angiotensin-mediated cardiac remodeling and fibrosis in mice with atrial fibrosis [37]. Confirming these results, Zhang M. et al. demonstrated that miR-27b improved cardiac function reducing the levels of the galectin-3 (encoded by the *LGALS3* gene), associated with cardiac remodeling both in vitro and in vivo hypertrophy models [38].

In addition, *mir22* found in our EVs derived from HL-1 cells was shown to modulate the expression of several genes involved in biological processes related to cardiac morphogenesis [39], as demonstrated in our bioinformatics analysis. This was also found in cardiomyocytes derived from murine embryonic stem cells [40]. It is worth noting that Chen et al. showed that pre-administration of miR-22 mimic in rats undergoing myocardial ischemia followed by 24 h of reperfusion inhibited cardiac myocyte apoptosis. MiR-22 inhibited cardiac myocyte apoptosis targeting the Caveolin-3 (Cav3) gene, an upstream negative regulator of endothelial nitric oxide synthase [41].

Our bioinformatics analysis also revealed the involvement of HL-1 EVs miRNAs in the development and differentiation processes related to the cardiovascular system. Our results showed that EVs also contain *mir378a*, whose presence was shown during the differentiation of human embryonic stem cells into cardiac cells. It is known that miR-378a targets the bone morphogenetic protein 2 (BMP2) gene, which is involved in early mesoderm induction and development and the regulation of cardiac function [42]. Moreover, it was found that miR-378a-3p might exert a cardioprotective effect against ischemic heart disease targeting Tripartite Motif 55 (TRIM55), which is involved in dilated cardiomyopathy, cardiomyocyte apoptosis, and differentiation [43]. Interestingly, miR-148a was able to induce myocardial differentiation in human bone mesenchymal stromal cells. MiR-148a targeting DNA methyltransferase 1 (DNMT1) promoted myocardial differentiation by modulating the methylation levels of GATA-binding protein 4 (GATA-4), a protein encoded by the GATA4 gene which is involved in myocardial differentiation [44].

Our HL-1 cardiac muscle cell-derived EVs also contained *mir30a*, *mir30d* and *mir30e*, three members of the miR-30 family. In line with our results, it has been shown that members of the miR-30 family (from miR-30a to miR-30e) are evolutionarily conserved and highly expressed in a mature heart [45,46]. Mir-30 targets the connective tissue growth factor (CTGF) gene involved in cell–matrix synthesis, which is required to maintain the functional integrity of heart tissue. Various authors have supposed that, by targeting CTGF, miR-30 modulated the differentiation of cardiomyocytes and fibroblasts [47,48]. Moreover, in compliance with our results, it was shown that miR-30d is secreted in EVs by cardiomyocytes and inhibits fibroblast proliferation and activation by directly targeting the gene that codes integrin α5 [49].

*Mir145a*, although very poorly expressed, is important for the maintenance of vascular smooth muscle cells phenotype by binding Kruppel-like factor 4 (Klf4), a positive regulator of proliferation and the phenotypic switch of these cells [50]. These findings demonstrate the role of miRNAs in modulating blood vessel diameter and contractility [51].

Some miRNAs are involved in differentiation processes and in the regulation of angiogenesis. Among these, *mir99b* is the second most expressed miRNA. MiR-99b is involved in the differentiation of human embryonic stem cells to endothelial cells. Indeed, endothelial cells infected with miR-99b transplanted into a mouse model of peripheral ischemia improved neoangiogenesis [52]. MiR-99b targets protein phosphatase 3 catalytic subunit alpha (*PP3CA*), also known as calcineurin Aα, involved in cardiac hypertrophy and electrical remodeling. In this way, miR-99b prevents calcineurin Aα from mediating cardiac hypertrophy and electrical remodeling [53]. Moreover, the involvement of miR-126a in the development of cardiomyocytes and the pro-angiogenic process was shown. Notably, miR-126a exerts these effects through the modulation of pro-angiogenetic factors and survival kinases such as ERK1/2 and Akt [54].

In our analysis, we found many differentially expressed miRNAs, several of which performed a regulatory function in blood vessel growth, such as *mir92-1*. Mir92-1 belongs to the miR-17-92 cluster. MiR-92a appears to target the mRNAs of several pro-angiogenic proteins, including the alpha5 integrin subunit (ITGA5), thereby regulating the growth of new vessels [55]. Moreover, it was shown that the miR-17-92 cluster targets the phosphatase and tensin homolog (PTEN) gene, identified as a regulator of the proliferation of cardiomyocytes [56].

Some of the miRNAs found in HL-1 EVs have already shown cardioprotective effects in experimental models. MiR-182 exerts cardioprotective effects in the rat model of heart failure. It was observed that the injection of adeno-associated virus-miRNA-182 inhibited cardiomyocyte apoptosis induced by heart injury through reduced levels of programmed cell death4 (PDCD4) and phosphofurin acidic cluster sorting protein 2 (PACS2) [57]. The miRNA profiles of endothelial cells revealed that specific miRNAs are enriched, including miR-21a and let-7b, among others [58,59]. It was demonstrated that the miR-21a-5p contained in mesenchymal stem cells exosomes is responsible for cardioprotection by synergistic suppression of multiple genes such as PTEN and PDCD4 [60]. Specifically, our bioinformatic analysis showed that *mirlet7b* and *mirlet7i*, despite being expressed less frequently compared to the most expressed miRNAs, targeted several genes related to cardiovascular development. In this regard, it was revealed that the let-7 family of miRNAs promoted cardiac maturation through the modulation of key genes metabolic such as insulin receptor substrate 2 (IRS2) and enhancer of zeste homolog 2 (EZH2) [61].

Our EVs contain other miRNAs, such as *mir183* and *mir25*. The miR-183 was detected in rat hypertrophic neonatal ventricular cardiomyocytes. It was shown that miR-183 targets the TIAM Rac1 associated GEF 1 (TIAM1) gene, a specific activator of the small axis GTPase Rac. TIAM1-Rac1 is also involved in the hypertrophy of neonatal rat cardiomyocytes [62]. Alongside this, miR-25 can regulate the proliferation of cardiomyocytes derived from human embryonic stem cells, modulating the F-Box and WD repeat domain containing 7 (FBXW7) gene, which is involved in the proteasome-mediated degradation of proteins such as cyclin E [63]. In an experimental model involving rats with ischemia/reperfusion injuries, Qin X.F. et al. demonstrated that miR-25 targets the Bcl-2-like protein 11 (BCL2L11) gene, which is involved in apoptosis [64].

It was demonstrated that the miR-99a targeting Smarca5 (lineage-specific transcription factors) regulated myocardiogenesis [65]. Mir-99a plays a cardioprotective role in post-infarction remodeling, improving cardiac function and survival in a mouse model of myocardial infarction [66]. MiR-10b directly targets the large tumor suppressor kinase 1 (LATS1) gene, which is associated with the hippo signaling pathway (involved in regulating the growth and death of cardiomyocytes) [67]. Instead, in an in vitro model of alcoholic cardiomyopathy, it was demonstrated that miR-186-5p can directly target the X-linked inhibitor of apoptosis (XIAP) gene (involved in apoptosis suppression) [68].

The role of mir451a in myocardiogenesis is poorly documented. However, it was observed that miR-451a reduced the level of matrix metalloproteinases (MMP) 2 and 3 in human cardiomyocytes during pathological stress [69].

Conversely, it has been shown that miR-26b induced cardiomyocyte differentiation by regulating both the canonical and non-canonical Wnt signaling by targeting both Gsk3β and Wnt5a [70]. In accordance with our results, Lozano-Velasco E. et al. also observed the presence of *mir125a* in HL-1 cardiomyocytes [71].

Li Y. et al. observed that treatment with exosomes secreted by bone mesenchymal stem cells carrying miR-301 could improve cardiac function and reduce myocardial infarction in rats with myocardial infarction. Mir-301 reduced the myocardial autophagy that down-regulated the expression of LC3-II, LC3-I and p62 (an autophagy-associated marker) [72]. In compliance with these results, in rats with myocardial infarction, the treatment of adipose-derived stromal cells-derived exosomes containing miR-93-5p showed a protective effect in rats with myocardial infarction, targeting Atg7 and Toll-like receptor 4 (TLR4) [73].

However, it is important to note that we used a cell line and that differences may exist when compared to cardiomyocytes. Kang I. S. et al. performed a characterization of the EVs isolated from human cardiac mesenchymal stromal cells and compared them with those isolated from human bone marrow-derived mesenchymal stem cells. The in vitro results of this study demonstrated that EVs derived from human cardiac mesenchymal stromal cells exhibited a potent angiogenic effect compared to EVs derived from bone marrow mesenchymal stem cells [74]. Moreover, Leitolis A. demonstrated that EVs isolated from the human heart induced proliferation and angiogenesis in endothelial cells, promoting wound healing, and reducing the adhesion of mesenchymal stem cells. Finally, it was demonstrated that human heart EVs were able to promote the re-cellularization of de-cellularized porcine heart valve leaflets [75].

Interestingly, some of the miRNAs that we found in HL-1-derived EVs were also found in other cell types. Among the miRNAs found in our EVs, *mir21a* and *mir22* were also found in bone marrow mesenchymal stem cells [60,76]. In these cells, these miRNAs, along with others, have been shown to be responsible for the beneficial effects of exosomes derived from these cells. MiRNA-21, along with miRNA-451, was also found in the exosomes derived from cardiac progenitor cells [77,78]. An in vivo study showed that miR-126, found in our EVs, was also expressed in exosomes derived from mesenchymal stem cells of adipose tissue and exhibited angiogenic effects [79]. On the contrary, it was demonstrated that cardiomyocytes produce many miRNAs (such as miR-1, miR-133a/b, miR-208a, and miR-49) that are specifically expressed in these cells [80]. These results demonstrate that, compared to EVs derived from other cells, cardiac EVs have a specific signature that underlines their origin. Therefore, according to our knowledge, only some of the miRNAs secreted by our EVs could be specific to the HL-1 cell line.

The HL-1 cells from which our EVs originated were in turn derived from AT-1 cells from an atrial tumor. AT-1 cells were grown in a transgenic mouse in which expression of the monkey virus large T antigen 40 (SV40) was targeted at its atrial cardiomyocytes via the promoter atrial natriuretic factor (ANF). The large T antigen binds to the retinoblastoma family (Rb family) of tumor suppressors and the tumor suppressor p53, contributing to the transformation [81]. Some miRNAs identified in our EVs are involved in processes that also play a role in tumor development, such as angiogenesis. Specifically, some of these miRNAs (such as miR-145a, miR-126 and mirR-92) regulate tumor growth, invasion, metastasis, and angiogenesis. However, they were reported to inhibit angiogenesis in the context of cancer [82,83,84,85]. This may suggest that the role of these miRNA in our study did not depend on the cancerous origin of the HL-1 cells.

## 5. Conclusions

This study provides evidence that EVs derived from HL-1 cardiac muscle cells contain several miRNAs, whose targets are involved in biological processes related to cardiac morphogenesis, development, differentiation, and angiogenesis. Consequently, we may suppose that the miRNA content of HL-1 EVs could be used to promote angiogenesis and the differentiation of mesenchymal stem cells in the field of cardiovascular regenerative medicine.

## Figures and Tables

**Table 1:** MiRNA expressed in HL-1 EVs.

MiRNA	Mean Expression	Standard Deviation
*mir143*	1390.67	499.34
*mir99b*	412.00	181.53
*mir22*	335.33	108.43
*mir182*	328.00	102.06
*mir378a*	192.00	70.31
*mir148a*	184.33	67.34
*mir27b*	155.67	59.18
*mir30a*	83.33	34.79
*mir21a*	75.67	30.24
*mir183*	67.00	30.64
*mir30e*	65.33	21.13
*mir25*	57.67	21.96
*mirlet7i*	57.33	18.88
*mir30d*	57.33	21.57
*mir92-1*	56.00	14.42
*mirlet7b*	40.33	13.87
*mir99a*	39.33	17.21
*mir10b*	38.00	18.73
*mir186*	31.00	11.53
*mir126a*	27.33	9.71
*mir301*	26.33	14.57
*mir125a*	22.67	8.39
*mir26b*	20.67	4.04
*mir451a*	17.00	6.24
*mir93*	16.67	4.73
*mir145a*	13.33	1.15

For each miRNA, the mean expression and standard deviation are related to the counts observed in the triplicate. The median value of the distribution is 57.33. *mir143*, *mir99b*, *mir22* and *mir182* are the upper outliers.

## Data Availability

The data presented in this study are openly available in the NCBI Sequence Read Archive at BioProject accession number PRJNA687733.

## Supplementary Materials

The following are available online at https://www.mdpi.com/2073-4409/10/2/273/s1, Figure S1: Whole Western blot analysis.

## References

[1] Vizoso F.J., Eiro N., Cid S., Schneider J., Perez-Fernandez R. (2017). Mesenchymal stem cell secretome: Toward cell-free therapeutic strategies in regenerative medicine. Int. J. Mol. Sci..

[2] Ratajczak M., Kucia M., Jadczyk T., Greco N., Wojakowski W., Tendera M., Ratajczak J. (2012). Pivotal role of paracrine effects in stem cell therapies in regenerative medicine: Can we translate stem cell-secreted paracrine factors and microvesicles into better therapeutic strategies?. Leukemia.

[3] Xia X., Chiu P.W.Y., Lam P.K., Chin W.C., Ng E.K.W., Lau J.Y.W. (2018). Secretome from hypoxia-conditioned adipose-derived mesenchymal stem cells promotes the healing of gastric mucosal injury in a rodent model. Biochim. Biophys. Acta (BBA) Mol. Basis Dis..

[4] Timmers L., Lim S.K., Arslan F., Armstrong J.S., Hoefer I.E., Doevendans P.A., Piek J.J., El Oakley R.M., Choo A., Lee C.N. (2008). Reduction of myocardial infarct size by human mesenchymal stem cell conditioned medium. Stem Cell Res..

[5] Nakamura Y., Miyaki S., Ishitobi H., Matsuyama S., Nakasa T., Kamei N., Akimoto T., Higashi Y., Ochi M. (2015). Mesenchymal-stem-cell-derived exosomes accelerate skeletal muscle regeneration. FEBS Lett..

[6] Sluijter J.P.G., Davidson S.M., Boulanger C.M., Buzas E.I., de Kleijn D.P.V., Engel F.B., Giricz Z., Hausenloy D.J., Kishore R., Lecour S. (2018). Extracellular vesicles in diagnostics and therapy of the ischaemic heart: Position paper from the working group on cellular biology of the heart of the european society of cardiology. Cardiovasc. Res..

[7] Valadi H., Ekstrom K., Bossios A., Sjostrand M., Lee J.J., Lotvall J.O. (2007). Exosome-mediated transfer of mrnas and micrornas is a novel mechanism of genetic exchange between cells. Nat. Cell Biol..

[8] Camussi G., Deregibus M.C., Bruno S., Grange C., Fonsato V., Tetta C. (2011). Exosome/microvesicle-mediated epigenetic reprogramming of cells. Am. J. Cancer Res..

[9] Zhang P., Yeo J.C., Lim C.T. (2019). Advances in technologies for purification and enrichment of extracellular vesicles. SLAS Technol..

[10] Wahid F., Shehzad A., Khan T., Kim Y.Y. (2010). Micrornas: Synthesis, mechanism, function, and recent clinical trials. Biochim. Biophys. Acta.

[11] Anglicheau D., Muthukumar T., Suthanthiran M. (2010). Micrornas: Small rnas with big effects. Transplantation.

[12] Hammond S.M. (2015). An overview of micrornas. Adv. Drug Deliv. Rev..

[13] Jakob P., Landmesser U. (2012). Role of micrornas in stem/progenitor cells and cardiovascular repair. Cardiovasc. Res..

[14] Claycomb W.C., Lanson N.A., Stallworth B.S., Egeland D.B., Delcarpio J.B., Bahinski A., Izzo N.J. (1998). Hl-1 cells: A cardiac muscle cell line that contracts and retains phenotypic characteristics of the adult cardiomyocyte. Proc. Natl. Acad. Sci. USA.

[15] Rajan T.S., Giacoppo S., Trubiani O., Diomede F., Piattelli A., Bramanti P., Mazzon E. (2016). Conditioned medium of periodontal ligament mesenchymal stem cells exert anti-inflammatory effects in lipopolysaccharide-activated mouse motoneurons. Exp. Cell Res..

[16] Diomede F., Merciaro I., Martinotti S., Cavalcanti M.F., Caputi S., Mazzon E., Trubiani O. (2016). Mir-2861 is involved in osteogenic commitment of human periodontal ligament stem cells grown onto 3d scaffold. J. Biol. Regul. Homeost. Agents.

[17] Diomede F., D’Aurora M., Gugliandolo A., Merciaro I., Orsini T., Gatta V., Piattelli A., Trubiani O., Mazzon E. (2018). Biofunctionalized scaffold in bone tissue repair. Int. J. Mol. Sci..

[18] Sharma S., Rasool H.I., Palanisamy V., Mathisen C., Schmidt M., Wong D.T., Gimzewski J.K. (2010). Structural-mechanical characterization of nanoparticle exosomes in human saliva, using correlative afm, fesem, and force spectroscopy. ACS Nano.

[19] Diomede F., D’Aurora M., Gugliandolo A., Merciaro I., Ettorre V., Bramanti A., Piattelli A., Gatta V., Mazzon E., Fontana A. (2018). A novel role in skeletal segment regeneration of extracellular vesicles released from periodontal-ligament stem cells. Int. J. Nanomed..

[20] Trubiani O., Bosco D., Di Primio R. (1994). Interferon-gamma (ifn-gamma) induces programmed cell death in differentiated human leukemic b cell lines. Exp. Cell Res..

[21] Diomede F., Zini N., Gatta V., Fulle S., Merciaro I., D’Aurora M., La Rovere R.M., Traini T., Pizzicannella J., Ballerini P. (2016). Human periodontal ligament stem cells cultured onto cortico-cancellous scaffold drive bone regenerative process. Eur. Cells Mater..

[22] Martin M. (2011). Cutadapt removes adapter sequences from high-throughput sequencing reads. Embnet. J..

[23] Dobin A., Davis C.A., Schlesinger F., Drenkow J., Zaleski C., Jha S., Batut P., Chaisson M., Gingeras T.R. (2013). Star: Ultrafast universal rna-seq aligner. Bioinformatics.

[24] Anders S., Pyl P.T., Huber W. (2015). Htseq--a python framework to work with high-throughput sequencing data. Bioinformatics.

[25] Ru Y., Kechris K.J., Tabakoff B., Hoffman P., Radcliffe R.A., Bowler R., Mahaffey S., Rossi S., Calin G.A., Bemis L. (2014). The multimir r package and database: Integration of microrna-target interactions along with their disease and drug associations. Nucleic Acids Res..

[26] Xiao F., Zuo Z., Cai G., Kang S., Gao X., Li T. (2009). Mirecords: An integrated resource for microrna-target interactions. Nucleic Acids Res..

[27] Chou C.H., Shrestha S., Yang C.D., Chang N.W., Lin Y.L., Liao K.W., Huang W.C., Sun T.H., Tu S.J., Lee W.H. (2018). Mirtarbase update 2018: A resource for experimentally validated microrna-target interactions. Nucleic Acids Res..

[28] Karagkouni D., Paraskevopoulou M.D., Chatzopoulos S., Vlachos I.S., Tastsoglou S., Kanellos I., Papadimitriou D., Kavakiotis I., Maniou S., Skoufos G. (2018). Diana-tarbase v8: A decade-long collection of experimentally supported mirna-gene interactions. Nucleic Acids Res..

[29] Ashburner M., Ball C.A., Blake J.A., Botstein D., Butler H., Cherry J.M., Davis A.P., Dolinski K., Dwight S.S., Eppig J.T. (2000). Gene ontology: Tool for the unification of biology. The gene ontology consortium. Nat. Genet..

[30] Thomas P.D., Campbell M.J., Kejariwal A., Mi H., Karlak B., Daverman R., Diemer K., Muruganujan A., Narechania A. (2003). Panther: A library of protein families and subfamilies indexed by function. Genome Res..

[31] Burridge P.W., Keller G., Gold J.D., Wu J.C. (2012). Production of de novo cardiomyocytes: Human pluripotent stem cell differentiation and direct reprogramming. Cell Stem Cell.

[32] White S.M., Constantin P.E., Claycomb W.C. (2004). Cardiac physiology at the cellular level: Use of cultured hl-1 cardiomyocytes for studies of cardiac muscle cell structure and function. Am. J. Physiol. Heart Circ. Physiol..

[33] Diomede F., Gugliandolo A., Cardelli P., Merciaro I., Ettorre V., Traini T., Bedini R., Scionti D., Bramanti A., Nanci A. (2018). Three-dimensional printed pla scaffold and human gingival stem cell-derived extracellular vesicles: A new tool for bone defect repair. Stem Cell Res. Ther..

[34] Hung W.T., Hong X., Christenson L.K., McGinnis L.K. (2015). Extracellular vesicles from bovine follicular fluid support cumulus expansion. Biol. Reprod..

[35] Deacon D.C., Nevis K.R., Cashman T.J., Zhou Y., Zhao L., Washko D., Guner-Ataman B., Burns C.G., Burns C.E. (2010). The mir-143-adducin3 pathway is essential for cardiac chamber morphogenesis. Development.

[36] Chinchilla A., Lozano E., Daimi H., Esteban F.J., Crist C., Aranega A.E., Franco D. (2011). Microrna profiling during mouse ventricular maturation: A role for mir-27 modulating mef2c expression. Cardiovasc. Res..

[37] Wang Y., Cai H., Li H., Gao Z., Song K. (2018). Atrial overexpression of microrna-27b attenuates angiotensin ii-induced atrial fibrosis and fibrillation by targeting alk5. Hum. Cell.

[38] Zhang M., Cheng K., Chen H., Tu J., Shen Y., Pang L., Wu W. (2020). Microrna-27 attenuates pressure overload-induced cardiac hypertrophy and dysfunction by targeting galectin-3. Arch. Biochem. Biophys..

[39] Huang Z.P., Wang D.Z. (2014). Mir-22 in cardiac remodeling and disease. Trends Cardiovasc. Med..

[40] Wang L., Cui Y., Tang M., Hu X., Luo H., Hescheler J., Xi J. (2014). Puerarin facilitates t-tubule development of murine embryonic stem cell-derived cardiomyocytes. Cell. Physiol. Biochem. Int. J. Exp. Cell. Physiol. Biochem. Pharmacol..

[41] Chen Z., Qi Y., Gao C. (2015). Cardiac myocyte-protective effect of microrna-22 during ischemia and reperfusion through disrupting the caveolin-3/enos signaling. Int. J. Clin. Exp. Pathol..

[42] Kim Y.Y., Min H., Kim H., Choi Y.M., Liu H.C., Ku S.Y. (2017). Differential microrna expression profile of human embryonic stem cell-derived cardiac lineage cells. Tissue Eng. Regen. Med..

[43] Tan J., Shen J., Zhu H., Gong Y., Zhu H., Li J., Lin S., Wu G., Sun T. (2020). Mir-378a-3p inhibits ischemia/reperfusion-induced apoptosis in h9c2 cardiomyocytes by targeting trim55 via the dusp1-jnk1/2 signaling pathway. Aging.

[44] Jiang C.K., Gong F. (2018). Mir-148a promotes myocardial differentiation of human bone mesenchymal stromal cells via DNA methyltransferase 1 (dnmt1). Cell Biol. Int..

[45] Forini F., Kusmic C., Nicolini G., Mariani L., Zucchi R., Matteucci M., Iervasi G., Pitto L. (2014). Triiodothyronine prevents cardiac ischemia/reperfusion mitochondrial impairment and cell loss by regulating mir30a/p53 axis. Endocrinology.

[46] Gutierrez-Escolano A., Santacruz-Vazquez E., Gomez-Perez F. (2015). Dysregulated micrornas involved in contrast-induced acute kidney injury in rat and human. Ren. Fail..

[47] Duisters R.F., Tijsen A.J., Schroen B., Leenders J.J., Lentink V., van der Made I., Herias V., van Leeuwen R.E., Schellings M.W., Barenbrug P. (2009). Mir-133 and mir-30 regulate connective tissue growth factor: Implications for a role of micrornas in myocardial matrix remodeling. Circ. Res..

[48] Chen L., Ji Q., Zhu H., Ren Y., Fan Z., Tian N. (2018). Mir-30a attenuates cardiac fibrosis in rats with myocardial infarction by inhibiting ctgf. Exp. Ther. Med..

[49] Li J., Salvador A.M., Li G., Valkov N., Ziegler O., Yeri A.S., Xiao C.Y., Meechoovet B., Alsop E., Rodosthenous R.S. (2020). Mir-30d regulates cardiac remodeling by intracellular and paracrine signaling. Circ. Res..

[50] Cordes K.R., Sheehy N.T., White M.P., Berry E.C., Morton S.U., Muth A.N., Lee T.H., Miano J.M., Ivey K.N., Srivastava D. (2009). Mir-145 and mir-143 regulate smooth muscle cell fate and plasticity. Nature.

[51] Albinsson S., Suarez Y., Skoura A., Offermanns S., Miano J.M., Sessa W.C. (2010). Micrornas are necessary for vascular smooth muscle growth, differentiation, and function. Arterioscler. Thromb. Vasc. Biol..

[52] Kane N.M., Howard L., Descamps B., Meloni M., McClure J., Lu R., McCahill A., Breen C., Mackenzie R.M., Delles C. (2012). Role of micrornas 99b, 181a, and 181b in the differentiation of human embryonic stem cells to vascular endothelial cells. Stem Cells.

[53] McGahon M.K., Yarham J.M., Daly A., Guduric-Fuchs J., Ferguson L.J., Simpson D.A., Collins A. (2013). Distinctive profile of isomir expression and novel micrornas in rat heart left ventricle. PLoS ONE.

[54] Shi H., Chen L., Wang H., Zhu S., Dong C., Webster K.A., Wei J. (2013). Synergistic induction of mir-126 by hypoxia and hdac inhibitors in cardiac myocytes. Biochem. Biophys. Res. Commun..

[55] Bonauer A., Carmona G., Iwasaki M., Mione M., Koyanagi M., Fischer A., Burchfield J., Fox H., Doebele C., Ohtani K. (2009). Microrna-92a controls angiogenesis and functional recovery of ischemic tissues in mice. Science.

[56] Chen J.H., Huang Z.P., Seok H.Y., Ding J., Kataoka M., Zhang Z., Hu X.Y., Wang G., Lin Z.Q., Wang S. (2013). Mir-17-92 cluster is required for and sufficient to induce cardiomyocyte proliferation in postnatal and adult hearts. Circ. Res..

[57] Zhou F., Fu W.D., Chen L. (2019). Mirna-182 regulates the cardiomyocyte apoptosis in heart failure. Eur. Rev. Med Pharmacol. Sci..

[58] Kuehbacher A., Urbich C., Zeiher A.M., Dimmeler S. (2007). Role of dicer and drosha for endothelial microrna expression and angiogenesis. Circ. Res..

[59] Suarez Y., Fernandez-Hernando C., Pober J.S., Sessa W.C. (2007). Dicer dependent micrornas regulate gene expression and functions in human endothelial cells. Circ. Res..

[60] Luther K.M., Haar L., McGuinness M., Wang Y., Lynch Iv T.L., Phan A., Song Y., Shen Z., Gardner G., Kuffel G. (2018). Exosomal mir-21a-5p mediates cardioprotection by mesenchymal stem cells. J. Mol. Cell. Cardiol..

[61] Kuppusamy K.T., Jones D.C., Sperber H., Madan A., Fischer K.A., Rodriguez M.L., Pabon L., Zhu W.Z., Tulloch N.L., Yang X.L. (2015). Let-7 family of microrna is required for maturation and adult-like metabolism in stem cell-derived cardiomyocytes. Proc. Natl. Acad. Sci. USA.

[62] Gong F.H., Chen X.L., Zhang Q., Xiao X.Q., Yang Y.S., Song B.J., Chao S.P., Cheng W.L. (2020). Microrna-183 as a novel regulator protects against cardiomyocytes hypertrophy via targeting tiam1. Am. J. Hypertens..

[63] Wang B., Xu M., Li M., Wu F., Hu S., Chen X., Zhao L., Huang Z., Lan F., Liu D. (2020). Mir-25 promotes cardiomyocyte proliferation by targeting fbxw7. Mol. Ther. Nucleic Acids.

[64] Qin X.F., Gao S.F., Yang Y.D., Wu L.L., Wang L.M. (2019). Microrna-25 promotes cardiomyocytes proliferation and migration via targeting bim. J. Cell Physiol..

[65] Coppola A., Romito A., Borel C., Gehrig C., Gagnebin M., Falconnet E., Izzo A., Altucci L., Banfi S., Antonarakis S.E. (2014). Cardiomyogenesis is controlled by the mir-99a/let-7c cluster and epigenetic modifications. Stem Cell Res..

[66] Li Q., Xie J., Li R., Shi J., Sun J., Gu R., Ding L., Wang L., Xu B. (2014). Overexpression of microrna-99a attenuates heart remodelling and improves cardiac performance after myocardial infarction. J. Cell. Mol. Med..

[67] Xie Y., Wang Q., Gao N., Wu F., Lan F., Zhang F., Jin L., Huang Z., Ge J., Wang H. (2020). Mircrorna-10b promotes human embryonic stem cell-derived cardiomyocyte proliferation via novel target gene lats1. Mol. Ther. Nucleic Acids.

[68] Liu Y., Yu B. (2019). Microrna1865p is expressed highly in ethanolinduced cardiomyocytes and regulates apoptosis via the target gene xiap. Mol. Med. Rep..

[69] Scrimgeour N.R., Wrobel A., Pinho M.J., Hoydal M.A. (2020). Microrna-451a prevents activation of matrix metalloproteinases 2 and 9 in human cardiomyocytes during pathological stress stimulation. Am. J. Physiol. Cell Physiol..

[70] Wang D., Liu C., Wang Y., Wang W., Wang K., Wu X., Li Z., Zhao C., Li L., Peng L. (2017). Impact of mir-26b on cardiomyocyte differentiation in p19 cells through regulating canonical/non-canonical wnt signalling. Cell Prolif..

[71] Lozano-Velasco E., Galiano-Torres J., Jodar-Garcia A., Aranega A.E., Franco D. (2015). Mir-27 and mir-125 distinctly regulate muscle-enriched transcription factors in cardiac and skeletal myocytes. BioMed Res. Int..

[72] Li Y., Yang R., Guo B., Zhang H., Zhang H., Liu S., Li Y. (2019). Exosomal mir-301 derived from mesenchymal stem cells protects myocardial infarction by inhibiting myocardial autophagy. Biochem. Biophys. Res. Commun..

[73] Liu J., Jiang M., Deng S., Lu J., Huang H., Zhang Y., Gong P., Shen X., Ruan H., Jin M. (2018). Mir-93-5p-containing exosomes treatment attenuates acute myocardial infarction-induced myocardial damage. Mol. Ther. Nucleic Acids.

[74] Kang I.S., Suh J., Lee M.N., Lee C., Jin J., Lee C., Yang Y.I., Jang Y., Oh G.T. (2020). Characterization of human cardiac mesenchymal stromal cells and their extracellular vesicles comparing with human bone marrow derived mesenchymal stem cells. BMB Rep..

[75] Leitolis A., Suss P.H., Roderjan J.G., Angulski A.B.B., da Costa F.D.A., Stimamiglio M.A., Correa A. (2019). Human heart explant-derived extracellular vesicles: Characterization and effects on the in vitro recellularization of decellularized heart valves. Int. J. Mol. Sci..

[76] Feng Y., Huang W., Wani M., Yu X., Ashraf M. (2014). Ischemic preconditioning potentiates the protective effect of stem cells through secretion of exosomes by targeting mecp2 via mir-22. PLoS ONE.

[77] Liwak-Muir U., Dobson C.C., Naing T., Wylie Q., Chehade L., Baird S.D., Chakraborty P.K., Holcik M. (2016). Erk8 is a novel hur kinase that regulates tumour suppressor pdcd4 through a mir-21 dependent mechanism. Oncotarget.

[78] Chen L., Wang Y., Pan Y., Zhang L., Shen C., Qin G., Ashraf M., Weintraub N., Ma G., Tang Y. (2013). Cardiac progenitor-derived exosomes protect ischemic myocardium from acute ischemia/reperfusion injury. Biochem. Biophys. Res. Commun..

[79] Luo Q., Guo D., Liu G., Chen G., Hang M., Jin M. (2017). Exosomes from mir-126-overexpressing adscs are therapeutic in relieving acute myocardial ischaemic injury. Cell. Physiol. Biochem. Int. J. Exp. Cell. Physiol. Biochem. Pharmacol..

[80] Li C., Pei F., Zhu X., Duan D.D., Zeng C. (2012). Circulating micrornas as novel and sensitive biomarkers of acute myocardial infarction. Clin. Biochem..

[81] Ahuja D., Saenz-Robles M.T., Pipas J.M. (2005). Sv40 large t antigen targets multiple cellular pathways to elicit cellular transformation. Oncogene.

[82] Ye D., Shen Z., Zhou S. (2019). Function of microrna-145 and mechanisms underlying its role in malignant tumor diagnosis and treatment. Cancer Manag. Res..

[83] Alhasan L. (2019). Mir-126 modulates angiogenesis in breast cancer by targeting vegf-a -mrna. Asian Pac. J. Cancer Prev. Apjcp.

[84] Haug B.H., Henriksen J.R., Buechner J., Geerts D., Tomte E., Kogner P., Martinsson T., Flaegstad T., Sveinbjornsson B., Einvik C. (2011). Mycn-regulated mirna-92 inhibits secretion of the tumor suppressor dickkopf-3 (dkk3) in neuroblastoma. Carcinogenesis.

[85] Olive V., Jiang I., He L. (2010). Mir-17-92, a cluster of mirnas in the midst of the cancer network. Int. J. Biochem. Cell Biol..

