# Supplementary material for: MicroRNA Profiling of HL-1 Cardiac Cells-Derived Extracellular Vesicles
Source: Cells. 2021 Jan 30;10(2):273. doi: 10.3390/cells10020273 (PMC7912193; doi:10.3390/cells10020273)
Supplement: Supplementary file 1 [file cells-10-00273-s001.pdf]

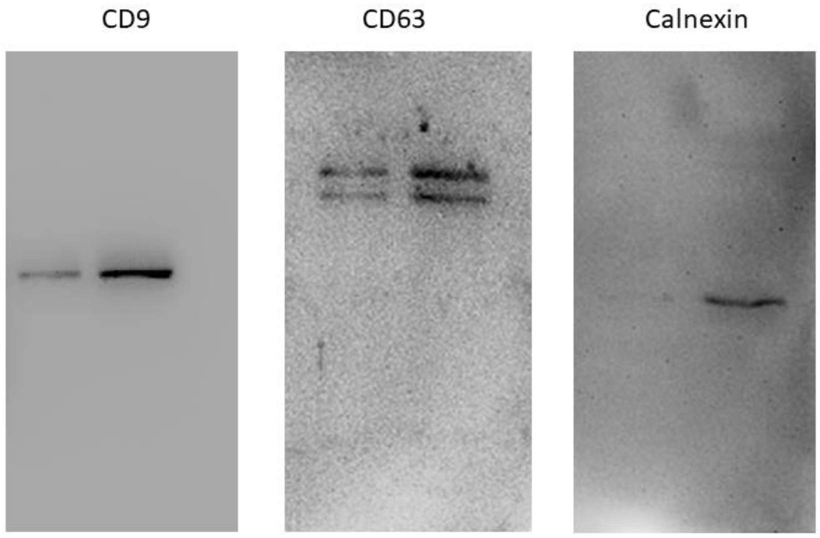

Figure S1. **Whole Western blot analysis.** Western Blot experiments performed on the EVs showed a positive expression for CD9 and CD63, specific membrane-associated proteins that are elevated in the EVs. Conversely, EVs exhibited a negative expression for Calnexin, excluding the impurity derived from other compartments to the EVs fractionally.
